# Role of Dicer1-Dependent Factors in the Paracrine Regulation of Epididymal Gene Expression

**DOI:** 10.1371/journal.pone.0163876

**Published:** 2016-10-03

**Authors:** Olivia Jerczynski, Nicolas Lacroix-Pépin, Eric Boilard, Ezequiel Calvo, Agathe Bernet, Michel A. Fortier, Ida Björkgren, Petra Sipilä, Clémence Belleannée

**Affiliations:** 1 Department of Obstetrics, Gynecology and Reproduction, Université Laval, CHU de Québec Research Center (CHUL), Quebec City, Quebec, Canada; 2 Department of Immunity and Infectious Diseases, Université Laval, CHU de Québec Research Center (CHUL), Quebec City, Quebec, Canada; 3 Endocrinology unit, CHU de Québec Research Center (CHUL), Quebec City, Quebec, Canada; 4 Department of Physiology, Institute of Biomedicine, University of Turku, Turku, Finland; 5 Turku Center for Disease Modeling, University of Turku, Turku, Finland; Institut de Pharmacologie Moleculaire et Cellulaire, FRANCE

## Abstract

Dicer1 is an endoribonuclease involved in the biogenesis of functional molecules such as microRNAs (miRNAs) and endogenous small interfering RNAs (endo-siRNAs). These small non-coding RNAs are important regulators of post-transcriptional gene expression and participate in the control of male fertility. With the knowledge that 1) Dicer1-dependent factors are required for proper sperm maturation in the epididymis, and that 2) miRNAs are potent mediators of intercellular communication in most biological systems, we investigated the role of Dicer1-dependent factors produced by the proximal epididymis (initial segment/caput)- including miRNAs- on the regulation of epididymal gene expression in the distal epididymis regions (*i*.*e*. corpus and cauda). To this end, we performed comparative microarray and ANOVA analyses on control *vs*. *Defb41*^*iCre/wt*^*;Dicer1*^*fl/fl*^ mice in which functional Dicer1 is absent from the principal cells of the proximal epididymis. We identified 35 and 33 transcripts that displayed significant expression level changes in the corpus and cauda regions (Fold change > 2 or < −2; p < 0.002), respectively. Among these transcripts, Zn-alpha 2-glycoprotein (*Azgp1*) encodes for a sperm equatorial protein whose expression in the epididymis of Dicer1 cKO mice is significantly increased compared to controls. In addition, 154 miRNAs, including *miR-210*, *miR-672*, *miR-191* and *miR-204*, showed significantly impaired biogenesis in the absence of Dicer1 from the principal cells of the proximal epididymis (Fold change > 2 or < −2; p < 0.01). These miRNAs are secreted via extracellular vesicles (EVs) derived from the DC2 epididymal principal cell line, and their expression correlates with target transcripts involved in distinct biological pathways, as evidenced by *in silico* analysis. Albeit correlative and based on *in silico* approach, our study proposes that Dicer1-dependent factors trigger- directly or not—significant genes expression changes in distinct regions of this organ. The paracrine control of functions important to post-testicular sperm maturation by Dicer1-dependent factors may open new avenues for the identification of molecular targets important to male fertility control.

## Introduction

Dicer1 is an RNase III enzyme involved in the canonical biogenesis of functional microRNAs (miRNAs) through trimming of miRNA precursors (pre-miRNA). Small (~22 nt) non-coding single-stranded miRNAs bind to target mRNAs and induce their degradation or inhibit their translation into proteins via RNA interference [1], for review [2, 3]. MicroRNAs are endogenously produced by all cells, following several steps of maturation. First, miRNAs are transcribed in the nucleus as long primary miRNA (pri-miRNA) transcripts by RNA polymerase II, and cleaved by the DiGeorge syndrome critical region 8 (DGCR8)/Drosha complex to form 70-nt-long stem-loop pre-miRNA. Following their export to the cytoplasm via Exportin 5, pre-miRNA undergo cleavage by Dicer1 to produce ~22-nt-long double-stranded miRNAs. One miRNA strand is finally assembled into the RNA-induced silencing complex (RISC) with Argonaute (AGO) proteins to interfere with the 3′-untranslated region (UTR) of target mRNA. This association results in the cleavage or translational repression of the target transcript. Since the expression of nearly 60% of human genes–and their respective biological pathways–is regulated at the post-transcriptional level by miRNAs, these small molecules are involved in the control of major pathological conditions (for review [3]), many of them being associated with male reproductive tract dysfunction leading to infertility [4–7].

MicroRNAs participate in a well-conserved mechanism of intercellular communication, as they can be released from cells and disseminated by extracellular fluids to reach and modify the functions of remote target cells [8]. These extracellular miRNAs (ex-miRNAs) can be found associated with different carriers such as high- and low-density lipoproteins [9, 10], ribonucleoproteins [11], or encapsulated and protected from RNAse assault in cell-derived extracellular vesicles (EVs) [12, 13]. Extracellular vesicles encompass a complex diversity of vesicles–including microvesicles and exosomes–that differ in terms of size, mode of secretion, lipid, protein and nucleic acid composition [8, 14]. Thus, ex-miRNAs transported in EVs belong to a category of paracrine messengers that stably exist in most biological fluids [15], including fluids found in the male reproductive system [16–18].

Similarly to other biological systems, male reproductive tract functions are regulated by Dicer1-dependent factors, including miRNAs, and by EVs secreted by distinct secretory organs such as the prostate and the epididymis [17, 19]. The epididymis governs the acquisition of sperm motility and oocyte binding ability and is a single long tubule, located downstream of the testis [20–22]. It is divided into three distinct regions: the proximal (initial/segment caput), median (corpus), and distal (cauda) regions. During epididymis transit, spermatozoa interact with the epididymal fluid, which is composed of different factors that are sequentially secreted/reabsorbed by distinct cell types of the surrounding epithelium. Principal cells are the main epithelial cells of the epididymis and are specialized in protein secretion via the classical exocytosis pathway and apocrine secretion of EVs, referred to as epididymosomes (for review [23]). Extracellular vesicles are capable of transferring proteins involved in different steps of the fertilization process to the sperm surface [24–27], as well as non-coding RNAs, including transfer RNA-derived fragments (tRFs) and miRNAs [28–30].

MicroRNAs are thought to be important in fertility since the double inactivation of *miR-34b/c* and *miR-449* miRNA clusters results in male infertility due to reduced sperm production and decreased sperm motility [6, 31]. In addition, the conditional deletions of the major enzymes involved in miRNA biogenesis (*i*.*e*. Dicer1 or Dgcr8) impair primordial germ cell development [32, 33] spermatogenesis, and sperm maturation [4, 5, 7, 34]. The importance of Dicer1 in epididymis homeostasis and sperm maturation has been shown in the *Defb41*^*iCre/wt*^*;Dicer1*^*fl/fl*^ mouse model. Epithelial dedifferentiation, abnormal lipid homeostasis, and sperm maturation defects are observed in these mice. For instance, sperm cells isolated from the distal epididymis of *Defb41*^*iCre/wt*^*;Dicer1*^*fl/fl*^ mice are predominantly immotile, and present with a decreased ability to bind and fertilize an oocyte [34].

Recognizing that 1) Dicer1-dependent factors are required for complete sperm maturation in the epididymis, and that 2) miRNAs are potent mediators of intercellular communication in most biological systems, we investigated the role of Dicer1-dependent factors, in the downstream paracrine regulation of epididymal gene expression. To this end, we used a combination of microarray approaches, technologies adapted to EV characterization and powerful bioinformatics tools along with the *Defb41*^*iCre/wt*^*;Dicer1*^*fl/fl*^ mouse model and DC2 cell lines. Thus, our study contributes to deciphering the intercellular communication pathways that support epididymal homeostasis and fertility.

## Materials and Methods

### Ethics

Our study on mouse epididymal samples was conducted in accordance with the requirements defined by the NIH Guide to animal experimentation. Animal experiments were approved by the ethical committee of the Institutional Review Board of the Centre Hospitalier Universitaire de Québec (CHUQ)(CPAC license #13-105-3, C. Belleannée).

### Mouse tissues

Epididymides from *Dicer1*^*fl/fl*^ (Control) and *Defb41*^*iCre/wt*^*;Dicer1*^*fl/fl*^ (Dicer1 conditional knock-out (Dicer1 cKO)) were used in this study. In this genetically modified mouse model, the enzyme Dicer1 has been conditionally inactivated in the principal cells of the initial segment/caput epididymidis by a Cre–Lox system under the control of the ß-defensin 41 promoter [5, 34]. Briefly, Dicer1 recombination was catalyzed by Defb41^iCre/wt^ before puberty (starting from postnatal day 12). The defensin-41 promoter was chosen as an epididymis-specific gene expressed only in the principal cells from the proximal epididymis. Epididymides from sacrificed mice were dissected into three main anatomical regions, *i*.*e*. the initial segment/caput, the corpus and the cauda. Paired tissue samples obtained from the same epididymal region were pooled and weighed. Tissues were directly snap frozen in liquid nitrogen and stored at −80°C until use to prevent RNA degradation. Spermatozoa were collected from adult C57Bl/6 mice (n = 4) for fluorescent immunostaining.

### Total RNA extraction and purification from mouse tissues

Total RNA was isolated from the initial segment/caput, the corpus and the cauda epididymal regions from three control and three Dicer1 cKO mice. Frozen tissues were powdered with a pestle and mortar on dry ice, and homogenized in RLT lysis buffer (Qiagen). Ribonucleic acid was purified with the RNeasy Mini Kit (Qiagen) according to the manufacturer's protocol, and potential genomic DNA contamination was eliminated with the RNase-free DNase set (Qiagen). Total RNA was eluted in RNAse-free water and its concentration was quantified with a NanoDrop 1000 microvolume spectrophotometer (Thermo Scientific). Ribonucleic acid quality was assessed with the Agilent RNA 6000 pico and Agilent RNA nano kits on a 2100 Bioanalyzer by the transcriptomic core facility of the CHUQ (Agilent). The RNA integrity numbers were in the range of 8.10 to 9.60 (S1 Fig). Samples were stored at −80°C until use.

### MicroRNA microarray profiling

Total RNA samples from the proximal epididymis (*i*.*e*. initial segment/caput) of three control and three Dicer1 cKO mice were used for microarray comparative analysis to identify Dicer1-dependent mature miRNAs produced by principal cells in this region. For each sample, 400 ng of total RNA were labeled with the FlashTag Biotin HSR Labeling Kit (Affimetrix, Santa Clara, USA) and hybridized to the GeneChip miRNA 4.0. Array (Affymetrix). This array covers 203 organisms, including human, rat and mouse, and contains 30,424 probe-sets, of which 1,908 correspond to murine mature miRNA sequences and 1,255 to miRNA precursor sequences. Probe-sets refer to nucleic sequences released in miRBase v.20.0 (http://www.mirbase.org/). Microarrays were scanned using the Affymetrix GeneChip Scanner 3000 7G and the Affymetrix GeneChip Command Console Software (Affymetrix, Santa Clara, CA), to produce the intensity files. The image data were analyzed with Expression Console software for quality control (Affymetrix) (S2 Fig) and extracted with GeneChip Operating Software (GCOS v 1.4; Affymetrix). CEL files were imported and analyzed with Partek Genomics Suite 6.5 software (Partek Incorporated), following a Robust Multiarray Analysis (RMA) background correction. All miRNA microarray data have been deposited in the Gene Expression Omnibus (GEO) repository database for public access (#GSE77139).

### Whole-transcript expression profiling

Total RNA samples from the *corpus* and the *cauda* epididymal regions of three controls and three Dicer1 cKO mice were used to determine the transcript signature found in these regions. Microarray analyses were performed by the CHUQ Gene Expression Platform (Quebec, Canada). Samples were hybridized to the GeneChip Mouse Gene 2.0 ST (Affymetrix). This array encompasses 35,240 transcript probe-sets, from three transcript data sources (*i*.*e*. RefSeq, Ensembl and lncRNA db). As described above, microarrays were scanned using the Affymetrix GeneChip Scanner 3000 7G and Affymetrix GeneChip Command Console software (Affymetrix, Santa Clara, CA), to produce the intensity files. The image data were analyzed with Expression Console Software for quality control (Affymetrix) (S2 Fig) and extracted with the GeneChip Operating Software (GCOS v 1.4; Affymetrix). CEL files were imported and analyzed with Partek Genomics Suite 6.5 software (Partek Incorporated). All gene expression microarray data have been deposited in the Gene Expression Omnibus (GEO) repository database for public access (#GSE77139).

### Bioinformatics analyses

Robust Multiarray Analysis (RMA) background correction was applied to all microarrays prior to expression analysis by using Partek’s software (Partek Incorporated, St Louis, MO, USA). Differentially expressed miRNAs or transcripts were analyzed by ANOVA pair-wise comparison between the distinct epididymal regions of Dicer1 cKO *vs*. Control mice. Principal component analysis (PCA), unsupervised hierarchical clustering, and volcano plots were performed with Partek Genomics Suite 6.5 software.

*In silico* study of biological pathways modified in Dicer1-cKO mice were performed using Ingenuity Pathway Analysis (IPA) software (http://www.ingenuity.com/), Gene Set Enrichment Analysis (GSEA, (http://www.broadinstitute.org/gsea/index.jsp), and the ClueGO Cytoscape program v2.2.2, 2015 (http://www.ici.upmc.fr/cluego/). Unless specified otherwise, stringency cut-off for gene set analysis was set to a fold-change > 1.5 and p < 0.01.

### Production/isolation of extracellular vesicles (EVs) from DC2 cell lines

DC2 cells are immortalized murine principal cells isolated from the caput epididymidis that were kindly provided by Professor Marie-Claire Orgebin-Crist (Vanderbilt University School of Medicine, Nashville, TN). DC2 cells were cultured in Iscove's Modified Dulbecco's Medium (IMDM) (Invitrogen, ON, Canada) supplemented with 5α-di-hydrotestosterone (DHT, 1 nM) and 10% fetal bovine serum (Invitrogen, ON, Canada) at 32.8°C as described previously [35]. Confluent monolayers of DC2 cells were washed twice with PBS prior to treatment. For fluorescent detection of EVs secreted from epididymal principal cells, DC2 cells were incubated for 30 min with 10 μM CellTracker Green CMFDA Dye (Life Technologies, ON, Canada) in IMDM without serum, and then washed again twice with PBS as previously described [36]. Cells were then incubated with 1 μM calcium ionophore with or without exosome-free serum for 30 min or 24 h. To produce exosome-free serum, FBS was filtered through a 0.22-μm-pore-size membrane (Sarstedt, QC, Canada) and centrifuged overnight at 120 000 × *g*. This was followed by another centrifugation for 80 min at 120 000 × *g* [37]. Efficiency of EV depletion was assessed by high-sensitivity flow cytometry (HS-FCM) and zetasizer as described below. After incubation, cell culture supernatants were collected and centrifuged for 10 min at 800 × *g* to eliminate cells and apoptotic bodies, then aliquoted and frozen at −80°C prior to analysis. For EV purification, the supernatant was filtered and centrifuged for 80 min at 120 000 × *g* and then washed with PBS and centrifuged again for 80 min at 120 000 × *g*. The pellet was then resuspended in 150 μl of PBS.

### Immunocytochemistry

For immunostaining, the DC2 cells were cultured on an 8-chamber slide (Lab-Tek, Nunc Inc. Naperville, Illinois), then washed twice with PBS and fixed in precooled (−20°C) 100% methanol solution for 2 min. Cells were then washed again twice with PBS and air-dried prior to storage at −20°C until use. The fixed cells were serially hydrated for 15 min with PBS, then treated with PBS pH 7.2 containing 1% SDS for 4 min at room temperature. The slides were washed twice with PBS for 5 min then incubated with PBS containing 1% (w/v) BSA (Sigma, St-Louis, MO) for 15 min. The fixed cells were incubated with FITC-conjugated anti-cytokeratin antibody (1:100, F3418, Sigma, St-Louis, MO) overnight at 4°C in a moist chamber. Cells were then washed twice in high salt PBS (PBS containing 2.7% (w/v) NaCl) for 5 min and then washed twice with PBS for 5 min. DAPI mounting medium (VectaShield, H-1200, Vector Laboratories, Burlingame, CA) was used prior to coverslip application and immunofluorescence-stained samples were examined with a Zeiss Axioskop 2 epifluorescence microscope (Carl Zeiss Canada).

### Characterization of extracellular vesicles (EVs) by high-sensitivity flow cytometry (HS-FCM) and zetasizer

Extracellular vesicles secreted from cultured DC2 cells were analyzed using highly sensitive technologies, such as HS-FCM and nanosizer, which were adapted for the detection of small particles.

#### HS-FCM

CMFDA-positive EVs were directly detected from cell culture supernatants with a FACS Canto II (BD Biosciences, CA, USA) equipped with powerful blue laser (100 mW) and a forward scatter (FSC) coupled to a photomultiplier tube (PMT) "small particles option" (FSC-PMT). This sensitive approach allows the detection of labelled EVs without performing a preliminary purification step. With the exception of Tween 20, all buffers used for flow cytometry were filtered through a 0.22-μm-pore-size membrane. In brief, 50 μl of cell culture supernatants were incubated for 30 min at room temperature, either alone or in the presence of Triton X-100 (0.05%), EDTA (50 μM) or Tween 20 (15%) as negative controls prior to antibody/probe labelling [36, 38]. Each antibody/probe was incubated in annexin V buffer alone, in the absence of cell culture supernatant to determine the background noise. EVs were characterized according to exposure of exosome-like vesicle markers such as CD9, and phosphatidylserine. For EV labelling, 2.5 μl of fluorescent V450-conjugated annexin V (BD Biosciences, CA, USA) and/or 0.25 μg of FITC-conjugated anti-CD9 antibody (Abcam, ON, Canada) were incubated for 30 min in annexin V buffer with the cell culture supernatant at room temperature in a final volume of 200 μl. For all conditions, 15-μm diameter polystyrene microspheres (Polyscience, PA, USA) were added to each tube, and 1,500 beads per sample, determined by fluorescence, were acquired for acquisition normalization, as previously described [39]. Flow cytometry detection was performed using a Fourier optical transformation unit. Data were analyzed with the FloJo program.

#### Zetasizer Nano-ZS

Size distribution of small particles was analyzed with the Zetasizer Nano-ZS (Malvern Instruments, Ltd., Malvern, United Kingdom) on culture cell supernatants or purified EVs as previously described [40]. This approach uses the Dynamic Light Scattering (DLS) system by measuring Brownian motion of small particles in suspension, which correlates with particle size. Phosphate buffered saline or cell culture medium without EVs were used as negative controls. The data represent the average of two sets of 12–17 measurements, performed at 4°C.

### Small RNA purification

Small-sized RNAs (< 100 nt), including miRNAs, were purified from DC2-derived EVs and caput epididymidis. After solubilization of the EV pellet or of the powdered-tissue in lysis buffer, small RNAs were purified using the mirVana TM miRNA Isolation Kit (Life Technologies) according to the manufacturer’s recommendation and following the procedure for small RNA enrichment. Small RNAs were eluted in 50 μl of RNAse-free water at 90°C. Small RNA concentration was determined using a NanoDrop 1000 microvolume spectrophotometer.

### Reverse transcription and quantitative real-time PCR (qRT-PCR)

The most significant changes detected in transcript and miRNA levels between Control and Dicer1 cKO mice were validated by qRT-PCR using two adapted methodologies.

#### qRT-PCR on transcripts

Real-time PCR quantification of transcripts differentially expressed in the *corpus* and *cauda* was performed to validate the cDNA microarray results. In brief, 200 ng of total RNA extracts were denatured with 2 μg of random primers and 10 mM of each dNTP for 5 min at 65°C and then transferred directly onto ice. After addition of First-Strand Buffer (Life Technologies), 0.2 μM dithiothreitol (Invitrogen), 40 units of RNase inhibitor (Invitrogen) and 200 units of SuperScript II Reverse Transcriptase (Life Technologies), the mixture was incubated for 10 min at 25°C, 60 min at 42°C and finally 15 min at 70°C. Quantitative RT-PCR was performed on reverse transcribed (RT) templates with specific primers by using the LightCycler FastStart SYBR Green I kit (Roche Diagnostics). As a first step, temperature gradients and standard curves were performed to determine the optimal annealing temperatures and to assess primer efficiencies for each primer set. Three to four points were used to plot the standard curve. For the qRT-PCR reaction, 0.5 μM of specific forward and reverse primers and 4 μl of cDNA samples were added to the SYBR Green I master mix (Roche). A negative control performed in absence of RT template (NTC) was included. Samples were incubated at 95°C for 10 min followed by 45 cycles of three amplification steps: 95°C for 10 s, the optimal primer-specific temperature for 10 s and 72°C for 30 s. Samples were denatured at 95°C for 10 s and then cooled to 65°C for 60 s at 20°C per second for melting-curve analysis. Fluorescence signals were continuously collected at 530 nm from 65°C to 95°C, with a temperature change rate of 0.2°C per second. Primers used in this study and their respective characteristics are listed in S1 Table. Each qRT-PCR reaction was performed in duplicate on three biological replicates and normalized to *Gapdh*. Amplified products were resolved on a 2% agarose gel and sequenced to certify specificity of amplification. Results are expressed as relative quantification values based on cycle threshold (Ct) comparison between the different samples, and were analyzed using the Pfaffl method to calculate fold inductions [41].

#### qRT-PCR on miRNAs

Quantitative RT-PCR of miRNAs differentially expressed in the initial segment/caput epididymidis was performed to validate miRNA microarray results, as previously described [42, 43]. In brief, specific miRNAs present in total RNA extracts were first reverse transcribed using a stem-loop primer. Ten to 15 ng of total RNA extract were denatured with 1 μM of the appropriate stem-loop primer, and 10 mM of each dNTP for 5 min at 65°C and then transferred directly onto ice. After addition of First-Strand Buffer (Life Technologies), 10 mM dithiothreitol (Invitrogen), 4 units of RNase inhibitor (Invitrogen) and 50 units of SuperScript II Reverse Transcriptase (Life Technologies), the mixture was incubated for 30 min at 16°C, followed by 60 cycles of: 30 s at 30°C, 30 s at 42°C and 1 s at 50°C. A final 5-min incubation at 85°C was performed to inactivate the reverse transcriptase. Quantitative RT-PCR was performed with 4 μl of cDNA samples added to 0.5 μM of forward and universal primer and 4 μl SYBR Green I master mix, for a total volume of 10 μl. A no-template control was included (NTC) in each PCR reaction. Samples were incubated at 95°C for 5 min followed by 42 cycles of two amplification steps: 95°C for 5 s and 60°C for 10 s. Samples were denatured at 95°C for 10 s and then cooled to 65°C for 60 s at 20°C per second for melting-curve analysis. Fluorescent signals were continuously collected at 530 nm from 65°C to 95°C. All the miRNAs primers used in this study are listed in S2 Table. The qPCRs were performed in duplicate on three biological replicates and normalized to Let7b expression. Amplified products were resolved on a 3.5% agarose gel to confirm the size of the amplified product. Results are expressed as relative quantification values based on cycle threshold (Ct) comparison between the different samples and were analyzed using the Pfaffl method to calculate fold inductions.

### Western-blot

Protein extraction from initial segment/caput, corpus and cauda epididymides from control (n = 3) and Dicer1 cKO (n = 3) mice was done mechanically on ice in RIPA buffer (150 mM NaCl, 50mM Tris, 0,1% SDS, 1% Triton, 0,5% deoxycolate, 1mM EDTA, pH 7,4). Cell debris was removed by centrifugation at 4^°^C for 15 minutes. Protein extracts were denatured and reduced by boiling in Laemmli sample buffer at 90^°^C for 5 minutes. According to the dynamic range of Zinc-alpha-2-glycoprotein (AZGP1, also known as ZAG) and β-actin detection (S3 Fig), 20μg of proteins were loaded on SDS-PAGE gel of 10% acrylamide and transferred onto cellulose membranes using the Trans-Blot Turbo system (Bio-Rad). Membranes were blocked in 5% milk in PBS containing 0,05% Tween 20 for 1 hour and then incubated overnight at 4^°^C with a rabbit polyclonal antibody against AZGP1 (Antibodies-online Inc. Atlanta, No. ABIN2707262)(1:500) in 5% milk in PBS 1X. After three washes in PBS with 0.05% Tween 20, membranes were incubated with a donkey anti- rabbit IgG conjugated to horseradish peroxidase for 1 h at room temperature. After three further washes, antibody binding was detected with the Western Lightning Plus-ECL reagent (PerkinElmer, Inc, Waltham, MA 02451) and the ChemiDoc MP Imaging System (Bio-Rad). Quantification of AZGP1 was performed by measuring the band volume intensity with ImageLab system (BioRad) and normalized on β-actin expression.

### Immunofluorescence on spermatozoa

Epididymal spermatozoa were collected from the cauda epididymidis by intraluminal perfusion with PBS [44] and treated for immunofluorescence as previously described [45]. After extensive washes in PBS, spermatozoa were fixed in Periodate-lysine-paraformaldehyde fixative (PLP) for 15 min, permeabilized with 2% Triton X-100 for 15 minutes, washed in PBS, and blocked for 30 min in 1% BSA/PBS. Spermatozoa were incubated overnight at 4^°^C with polyclonal rabbit antibody against AZGP1 or purified rabbit IgG (negative control), diluted to 5 mg/ml and for 30 minutes at room temperature with Alexafluor 488-conjugated goat anti-rabbit antibody (Jackson ImmunoResearch Laboratories), diluted to 2 mg/ml. Peanut agglutinin (PNA) -FITC (Sigma) was added at final concentration of 50μg/ml to label the sperm acrosomal region. After washes in PBS, a drop of sperm suspension was deposited on surperfrost slides (Fisherbrand, Pittsburgh, PA) and counterstained with Vectashied mounting medium containing DAPI. Image acquisition was performed on Olympus IX81-ZDC fluorescent microscope equipped with a Spinning Disc confocal system (Quorum Technologies, Guelph, ON) and Metamorph NX software.

### Immunohistochemical staining

Cryosections of 10 μm were prepared from paraffin-embedded epididymal tissues from control (n = 3) and Dicer1 cKO (n = 3) mice. Following deparaffinization, endogenous peroxidase activity was quenched with 3% H_2_O_2_ (v/v) in methanol for 10 minutes. Sections were washed for 5 minutes in 95% ethanol and 5 minutes in PBS. Non-specific binding sites were blocked with 10% goat serum in PBS for 1 h. Antibodies against AZGP1 were diluted (1:50) in DAKO (Dako North America, Inc.) and applied overnight at 4^°^C. For control sections, PBS replaced the primary antibodies. Sections were subsequently incubated with biotinylated goat anti-rabbit antibody for 60 min, and with ABC elite reagent (Vector Laboratories, Inc. Burlingame, Ca) for 30 min. Immunostaining was revealed using 3-amino-9-ethylcarbazole (AEC). Mayer’s haematoxylin solution was used for counterstaining, and mounted under cover slips using an aqueous mounting medium (Sigma). Slides were observed under a Zeiss Axioskop2 Plus microscope linked to a digital camera from Qimaging. Images were captured using the QCapture Pro (Qimaging Instruments).

### Statistical analysis

#### HS-FCM

Data were analyzed by one-way ANOVA with Dunnett’s multiple comparison test as post-hoc test with 95% confidence intervals using the GraphPad Prism 5 program. Data are presented as the mean ± SEM. Each experiment was repeated at least three times.

#### qRT-PCR on cDNA

Data were analyzed by multiple t-tests corrected for multiple comparisons using the Holm-Sidak method with 95% confidence intervals using the GraphPad Prism 5 program. Data are presented as the mean ± SEM. Each experiment was repeated on three biological samples/group.

#### qRT-PCR on miRNAs

Data were analyzed by unpaired t-test with 95% confidence intervals using the GraphPad Prism 5 program. Each experiment was repeated on three biological samples/group.

## Results

### Identification of Dicer1-dependent microRNAs in principal cells from the proximal epididymidis

Principal cells of the epididymis participate in the secretion of EVs, referred to as epididymosomes (For review, [17]). These EVs transport a large diversity of small non-coding RNAs, including miRNAs, and participate in extracellular communication mechanisms between somatic cells and the maturing sperm cells [28, 30, 46]. In order to identify Dicer1-dependent miRNAs that are mainly derived from principal cells of the proximal epididymidis, we performed a comparative microarray analysis of the miRNA profile found in Dicer1 cKO *vs*. control mice.

**Fig 1:**
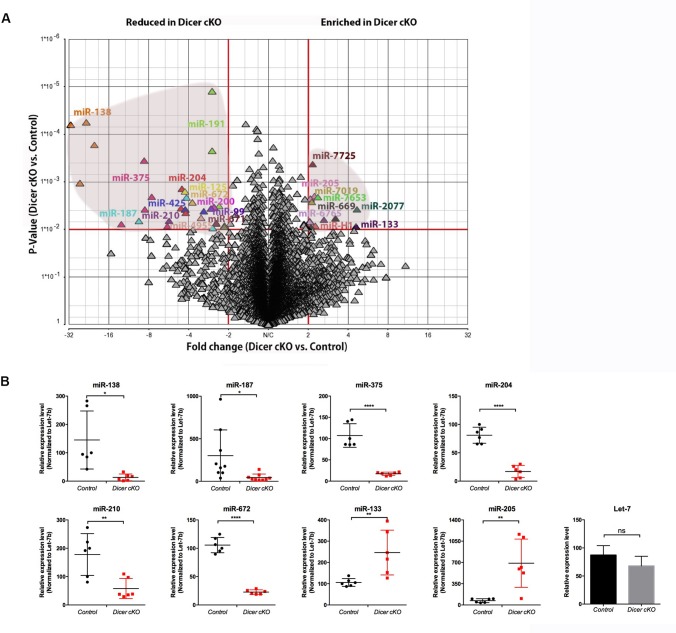
miRNA signature change in the proximal epididymis region (*i*.*e*. initial segment/caput) of Dicer1 cKO mice. **(A)** The different miRNA profiles of Dicer1 cKO and control mice are plotted on the volcano plot according to two-way ANOVA parameters, *i*.*e*. p-value and fold-change. Only miRNAs with a fold change above 2 and a p-value below 0.01 (red lines) are annotated. **(B)** Changes in mature miRNA production in Dicer1 cKO were assessed by qRT-PCR. Data represent experimental duplicates on n = 3 biological samples per group and are normalized to Let-7b expression. ns: non significant, *p < 0.05, **p < 0.01, ***p < 0.001, ****p < 0.0001.

Analysis of variance analysis performed on these datasets indicates that the miRNA signature is significantly modified in the Dicer1 cKO mouse, with decreased detection of 114 mature miRNAs and increased detection/enrichment of 40 mature miRNA sequences (fold change > 2 or < −2 and p-value < 0.01) (Fig 1A, S3 Table). Most of these miRNAs are highly conserved as 91% are positively detected by the miRNA probe-sets from more than four distinct organisms. Among murine miRNAs that display highly significant changes (fold change > 2 or < −2 and p-value < 0.01), nine mature miRNAs (*miR-138-5p*, *miR-204-3p*, *miR-425-5p*, *miR-672-5p*, *miR-99b-3p*, *miR-191-5p*, *miR-200c-3p*, *miR-671-3p*, and *miR-652-3p*) were detected with a lower intensity level in Dicer1 cKO mice compared with the control, whereas five miRNAs (*miR-205-5p*, *miR-7019-5p*, *miR-7653-5p*, *miR-466-5p* and *miR-669-5p*) were slightly enriched in the Dicer1 cKO mice (Table 1). The mature miRNA most affected by Dicer1 inactivation in principal cells is *miR-138-5p*, as its expression intensity is decreased by a factor of 26 with an intensity level below the threshold of detection in Dicer1 cKO mice.

Contrary to the mature miRNA species, the expression of murine stem-loop precursors is not significantly modified in Dicer1 cKO mice *vs*. control (fold change > 1.5 or < −1.5, p-value < 0.01), with the exception of *pre-miR-375*, which shows a 1.7-fold downregulation (S3 Table).

**Table 1:** List of murine mature miRNAs displaying a reduced or increased intensity of detection in Dicer1 cKO *vs*. control (Ctrl) mice.

	MicroRNA	Fold-Change (Dicer1 cKO *vs*. Ctrl)	P-value (Dicer1 cKO *vs*. Ctrl)
↑ **in Dicer1 cKO**	mmu-miR-138-5p	-26,449	0,00106
	mmu-miR-204-3p	-4,49045	0,00140
	mmu-miR-425-5p	-4,24086	0,00380
	mmu-miR-672-5p	-3,74912	0,00271
	mmu-miR-99b-3p	-2,71028	0,00362
	mmu-miR-191-5p	-2,65916	0,00001
	mmu-miR-200c-3p	-2,57172	0,00340
	mmu-miR-671-3p	-2,47448	0,00744
	mmu-miR-652-3p	-2,14466	0,00837
↓ **in Dicer1 cKO**	mmu-miR-205-5p	2,07404	0,00226
	mmu-miR-7019-5p	2,12703	0,00265
	mmu-miR-7653-5p	2,3651	0,00209
	mmu-miR-466m-5p	3,2093	0,00585
	mmu-miR-669m-5p	3,2093	0,00585

We used qRT-PCR to assess the detection level of eight mature miRNA candidates whose expression intensity is changed in the proximal epididymidis of Dicer1 cKO compared with control mice (Fig 1B). Among these, six miRNAs (*miR-138*, *miR-187*, *miR-375*, *miR-204*, *miR-210* and *miR-672*) are consistently and significantly detected at a lower intensity level (*i*.*e*., elevated Cq value or Cq values below the detection threshold) in Dicer1 cKO *vs*. control mice. At the opposite, *miR-133* and *miR-205* display a significant increased expression level in Dicer1 cKO compared to control group.

### *miR-210*, *miR-672*, *miR-191* and *miR-204* are secreted from principal cells via extracellular vesicles

**Fig 2:**
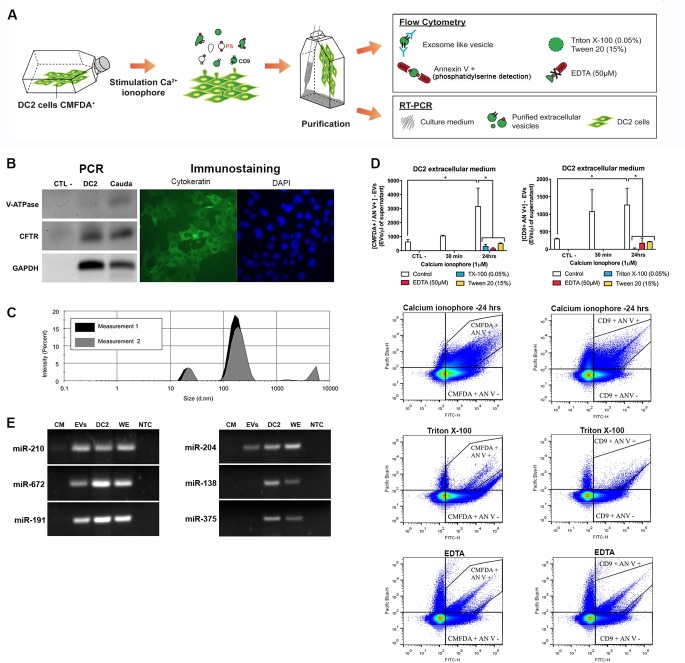
Secretion of Dicer1-dependent miRNAs from DC2 cells via extracellular vesicles (EVs). **(A)** Schematic view of DC2 derived EVs analysis. DC2 cells were cultured, labelled with CMFDA dye and stimulated with calcium ionophore to induce EVs production. EVs were characterized according to i) their surface antigens (*e*.*g*. CD9 marker, phosphatidylserine (PS) by flow cytometry, and to ii) their miRNA content by RT-PCR. **(B)** Detection of *Cftr* (principal cell marker) and *Atp6v1b1* (B1 V-ATPase subunit, clear cell marker) in cauda epididymis and DC2 cell line extracts by end-point PCR. Immunostaining for cytokeratin (green) on DC2 cells. Nuclei were counterstained with DAPI. **(C)** Size distribution of EVs released from DC2 cells in culture measured in a Zetasizer. This plot is representative of acquisitions performed twice on three distinct biological replicates. **(D)** Detection of CMFDA-positive EVs released from CMFDA-labeled DC2 cells by High-Sensitivity Flow Cytometry (HS-FCM). Supernatants from DC2 cells stimulated or not with 1 μM calcium ionophore for 30 min or 24 h were analyzed by HS-FCM for EV detection after annexin V (phosphatidylserine detection) and CD9 labeling. Controls include Triton X-100 0.05% that solubilizes most membranous particles, and EDTA 50 μM that inhibits annexin V labelling. **(E)** Detection of Dicer1-dependent miRNAs in DC2-derived EVs by end-point PCR in cells, whole tissue, and extracellular medium extracts. CM: culture medium; EVs: purified extracellular vesicles; DC2: culture medium-free DC2 cells; WE: whole epididymis; NTC: no-template control.

Extracellular vesicles secreted by epithelial cells of the epididymis are heterogeneous in terms of size, nucleic acid content, lipid composition, and marker protein exposure [27, 30, 47, 48]. Whereas a significant amount of epididymal fluid can be collected from the intraluminal compartment of large mammalian species by microperfusion, epididymal fluid retrieval from the proximal mouse epididymis is challenging and the yield is insufficient for downstream analyses. In our study we used the previously characterized DC2 epididymal cell line [35, 49] to 1) isolate and “characterize” EVs secreted by principal cells of the proximal epididymis and to 2) determine the route of secretion of Dicer1-dependent miRNAs (Fig 2A).

As shown by immunofluorescent staining (Fig 2B), DC2 cells are positive for cytokeratin protein, a marker of epithelial cells. In addition, these cells express the cystic fibrosis transmembrane conductance regulator (*Cftr*) principal cell marker and are negative for the B1 subunit of the V-ATPase (*Atp6v1b1*) clear cell marker (Fig 2B)[49]. This confirms that DC2 cells maintain their differentiated cell features under the culture conditions used in our study. As assessed by zetasizer analysis, the size distribution of particles released from DC2 cells after 24 h in culture falls between the range of 90–400 nm, with a mean peak at 192.2 ± 32.06 (diameter nm ± standard deviation) (Fig 2C). This size distribution aligns with the size range previously assessed by electron microscopy for epididymosomes collected from epididymal tissue [48]. In order to further define the properties of the small particles released from DC2 cells, we used HS-FCM to examine the presence of specific EV markers (*i*.*e*. phosphatidylserine exposure and CD9 exosomal marker detection) and determined their membrane susceptibility to detergents (Fig 2A and 2D). Since HS-FCM is a highly-sensitive detection method, we pre-stained DC2 cells with CMFDA Cell Tracker Green in order to limit the detection of non-specific particles. In addition, all experiments were performed on DC2 cells cultured with medium containing EV-depleted FBS (refer to Material and Methods section). In the non-stimulated control condition, the concentration of CMFDA/annexin V-positive EVs and CMFDA/CD9-positive EVs was approximately 700 EVs and 350 EVs per μl of cell culture supernatant (Fig 2D, top panel). Both EV subpopulation concentrations significantly increased after 24 h of treatment in the presence of 1 μM calcium ionophore, with fold increases of 4.2 and 3.5, respectively. Of importance was the observance of 90–95% cell viability after 24 h of treatment with the calcium ionophore (not shown), which suggests that apoptotic bodies were not major components measured in DC2 cell culture supernatants. In addition, EV solubilization by detergent (Triton X-100, 0.05% and Tween 20, 15%) and calcium chelation by EDTA, which inhibits annexin V binding, significantly decreased CMFDA/annexin V-positive and CMFDA/CD9-positive EV concentration.

We next verified the susceptibility of Dicer1-dependent miRNAs to be released from principal cells via EVs. To this end, we isolated EVs derived from DC2 cells, and collected medium-free DC2 cells. The respective miRNA content of these samples was analyzed by end-point PCR (Fig 2E). Among Dicer1-dependent miRNAs investigated (Fig 1), *miR-138* and *miR-375* were detected in both DC2 cell extracts and whole epididymis control tissue, whereas these miRNAs were undetected in EV extracts. Conversely, *miR-210*, *miR-672* and *miR-191* and *miR-204* were present in both cell and EV extracts, suggesting the presence of a selective mechanism of miRNA secretion from principal cells of the epididymis.

### Gene expression pattern is altered in the corpus and cauda epididymidis from Dicer1 cKO mice

**Fig 3:**
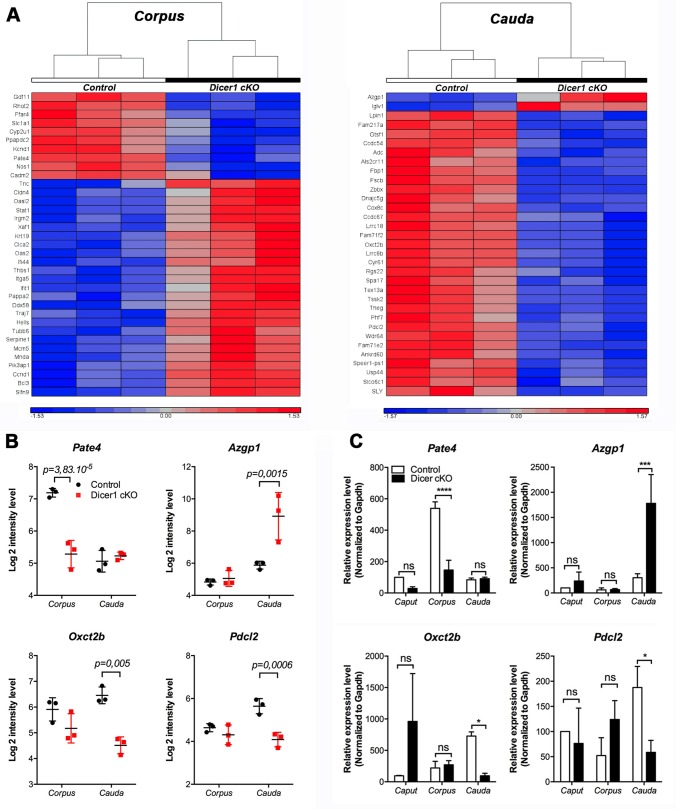
Impact of Dicer1-dependent factors on epididymal gene expression. **(A)** Transcripts with the most significantly altered expression in the corpus (left) or the cauda (right) epididymidis of the Dicer1 cKO mouse model are displayed on heat maps. Significance threshold: fold change > 2 or < −2, p-value < 0.002. n = 3 mice per group. **(B)** Relative Log 2 intensity levels of Prostate and testis expressed 4 (*Pate4*), Zinc-alpha-2-glycoprotein (*Azgp1*), Succinyl-CoA:3-ketoacid coenzyme A transferase 2B (*Oxct2b*), and Phosducin-like protein 2 (*Pdcl2*) in the corpus and cauda epididymidis of Dicer1 cKO and control mice after microarray analyses on n = 3 biological samples per group. **(C)** Expression changes of *Pate4*, *Azgp1*, *Oxct2b and Pdcl2* were validated by qRT-PCR in the caput, corpus and cauda regions from control and Dicer1 cKO mice. qRT-PCR data shown as means and standard deviations of results obtained from n = 3 biological samples per group after normalization to *Gapdh* expression. Unpaired t-test corrected for multiple comparison using the Holm-Sidak method. ns: non significant, *p<0.05, ***p < 0.001, ****p < 0.0001.

Segmented gene expression in the epididymis is regulated by several factors, including hormones, transcription factors and paracrine factors [21, 50]. In this study, we provide evidence that the production of some miRNAs is reduced in the Dicer1 cKO mouse model, and that epididymal principal cells from the proximal epididymis secrete some of these miRNA *in vitro*. To assess whether Dicer1-dependent factors, including miRNAs, could act as paracrine factors in the control of epididymal gene expression, we performed a comparative microarray analysis on gene expression patterns found in the corpus and cauda regions of Dicer1 cKO *vs*. control mice. Segment-specific expression of transcripts (*e*.*g*. members of the Defensin family) and miRNAs (*e*.*g*. *miR-204-5p and miR-196)* along the epididymis of control and Dicer1-cKO mice validated the purity of the tissue analysed (S4 Fig). Analysis of variance performed on these datasets indicates that the expression level of 141 genes is significantly changed (p < 0.001) in the distal regions of the Dicer1 cKO epididymis (S4–S6 Tables). Among the significantly changed genes, 35 up- and down- regulated genes (25 and 10, respectively) in the corpus region (Fig 3A, left panel), and 33 up- and down-regulated genes (2 and 31, respectively) in the cauda epididymidis of Dicer1 cKO are displayed (Fig 3A, right panel). Of importance is that none of the most significant changes identified by ANOVA overlap between the corpus and the cauda epididymis, suggesting that these two epididymal regions respond distinctly to Dicer1-dependent factors. *Pate4*, *Azgp1*, *Pdcl2 and Oxct2b* are four genes encoding the prostate and testis expressed 4 protein, the Zn-alpha 2-glycoprotein, Phosducin-like protein 2 and Succinyl-CoA:3-ketoacid coenzyme A transferase 2B, respectively. According to microarray analysis, these genes display a significant fold change in expression that has been validated by qRT-PCR (Fig 3B and 3C).

**Fig 4:**
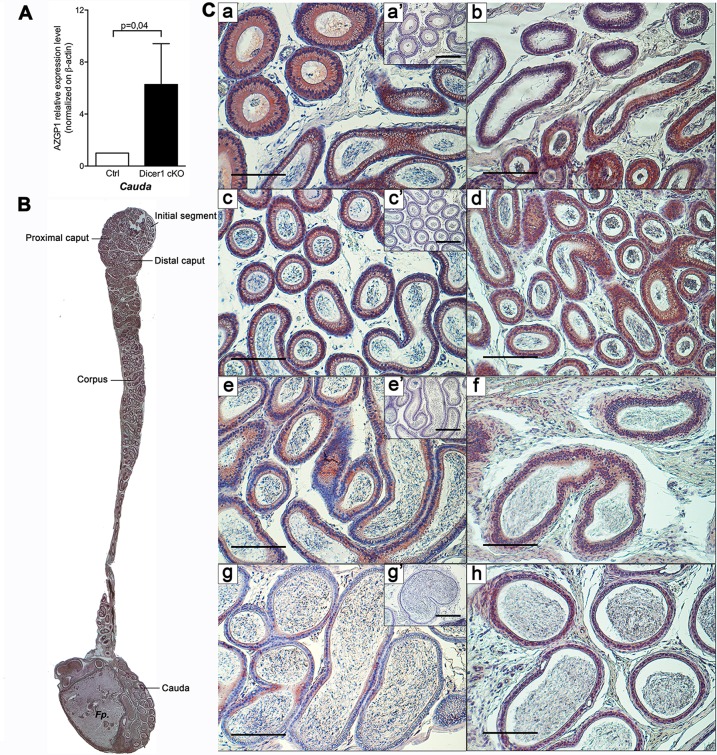
Protein Expression level of Zinc-alpha-2-glycoprotein (AZGP1) in the epididymis of Dicer1 cKO and control mice. **(A)** Relative quantification of AZGP1 protein in the cauda epididymis of Dicer1 cKO and control mice by western blot. Statistical significance was assessed by Student t-test from n = 3 replicates per group. **(B)** Longitudinal section of a mouse epididymis from a Dicer1 cKO mouse stained by immunohistochemistry for AZGP1. Image taken at 2.5 X. *Fp*: Fat pad. **(C)** Immuno-detection of AZGP1 on the proximal caput (a, b), distal caput (c, d), corpus (e, f) and cauda (g, h) epididymidis of control (a, c, e, g) and Dicer1 cKO (b, d, f, h) mice. Bar = 200 μm. Negative controls in absence of primary antibody are shown in insets (a’, c’, e’, g’). Bar = 250 μm.

**Fig 5:**
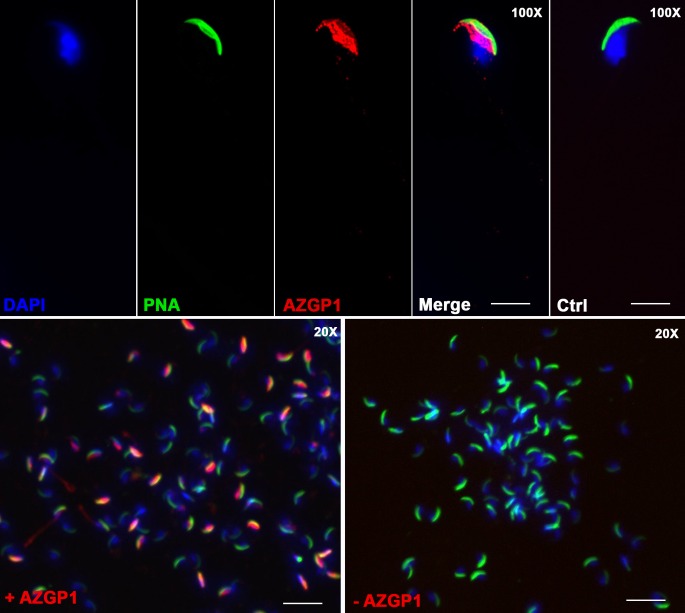
Localization of Zinc-alpha-2-glycoprotein (AZGP1) on spermatozoa from wild type mice. Sperm acrosomal region and nuclei were labelled with Peanut agglutinin lectin (PNA) and DAPI, respectively. Pictures were taken at 100X (top) and 20X (bottom) after immunostaining in the presence (+ AZGP1) or absence (Ctrl,—AZGP1) of antibody against AZGP1. Bars = 5 μm (top) and 20 μm (bottom).

The consequence of Dicer1 invalidation on AZGP1 protein expression was assessed by western-blot and immunohistochemistry in control and Dicer1 cKO mice (Figs 4 and 5). A unique band corresponding to the glycosylated form of AZGP1 (48 KDa [51]) was detected in mouse epididymal tissue extracts by western-blot (S3 Fig). Protein quantification indicated a significant six-fold increase of AZGP1 detection from the cauda epididymidis of Dicer1 cKO compared to control mice (Fig 4A). Protein expression of AZGP1 displayed a regionalized pattern of expression as evidenced by immunohistochemistry performed on longitudinal sections of control and Dicer1 cKO mouse epididymides (Fig 4B and 4C). For instance AZGP1 is highly expressed in the initial segment/caput epididymis, in apparently all cell types of the epithelium in control and Dicer1 cKO mice (Fig 4B and 4C_a,b_), and is undetectable in negative controls (Fig 4C_a’-d’_). While its expression drastically decreases in the cauda epididymis from control mice, its remains at a high expression level in the cauda epididymidis of Dicer1 cKO mice (Fig 4B and 4C_g,h_), which is in agreement with AZGP1 quantification measured by western-blot.

While AZGP1 is detected at a high level within the epididymal epithelium (Fig 4), this secreted glycoprotein might also interact with the maturing sperm cells in the epididymis. In order to assess this possible interaction, we detected AZGP1 by immunofluorescence on spermatozoa from the cauda epididymis of wild-type mice (Fig 5). A strong signal was observed for AZGP1 on the sperm equatorial segment, below the PNA-stained acrosomal region (Fig 5, top panel), and was absent in the isotype control condition (Fig 5, Ctrl). As assessed by confocal microscopy on low magnification pictures (Fig 5, bottom panel + AZGP1), the proportion of spermatozoa positive for AZGP1 is between 85–95%.

### *In silico* analysis of gene expression changes observed in the distal epididymidis of Dicer1 cKO mice

According to *in silico* target prediction, several Dicer1-dependent miRNAs could potentially bind to and regulate target mRNA whose expression in significantly modified in Dicer1 cKO mice (Table 2). For instance, among Dicer1-dependent miRNAs, miR-210, miR-204, miR-672, miR-191 and miR-296 are predicted to regulate 13 distinct transcripts whose expression is significantly modified in the corpus or cauda epididymidis of Dicer1 cKO mice. These transcripts encode proteins involved in lipid metabolism (*e*.*g*. Free fatty acid receptors 4/5, AZGP1), cytoskeleton formation (*e*.*g*. Cytokeratin 19 and Beta-tubulin), calcium transportation (*e*.*g*. PATE4, Calcium-activated chloride channel regulator 2), and transcription factors (*e*.*g*. B-cell lymphoma 3 protein, Myeloid cell nuclear differentiation antigen).

**Table 2:** Association of target genes predicted to be regulated by miRNAs candidates.

miRNA candidates	Predicted target genes	Complete gene name	Software		
			Targetscan	Microcosm	IPA*
mmu-miR-191-3p	*Clca2*	Calcium-activated chloride channel regulator 2	x		
mmu-miR-191-3p	*Prmt8*	Protein arginine N-methyltransferase 8		x	
mmu-miR-191-3p	*Mnda*	Interferon-activable protein 205-B		x	
mmu-miR-204-5p	*Pate4*	Prostate and testis expressed protein 4			x
mmu-miR-204-5p	*Krt19*	Keratin, type I cytoskeletal 19	x		
mmu-miR-204-5p	*Ffar4*	Free fatty acid receptor 4	x		
mmu-miR-210-5p	*Pate4*	Prostate and testis expressed protein 4	x		
mmu-miR-210-5p	*Lrrc18*	Leucine-rich repeat-containing protein 18	x		
mmu-miR-210-5p	*Bcl3*	B-cell lymphoma 3 protein		x	
mmu-miR-210-5p	*Tubb6*	Tubulin beta-6 chain		x	
mmu-miR-296-3p	*Azgp1*	Zinc-alpha-2-glycoprotein		x	
mmu-miR-672-3p	*Ffar4*	Free fatty acid receptor 4	x		
mmu-miR-672-3p	*Mcm5*	DNA replication licensing factor MCM5	x		
mmu-miR-672-3p	*Theg*	Testicular haploid expressed gene protein	x	x	

Only miRNAs and transcripts displaying significant expression changes (P<0.02 and P<0.05, respectively) in Dicer1-cKO mice compared to Control are shown. The distinct softwares used for *in silico* target prediction are indicated.

* *In silico* prediction performed by using the Ingenuity Upstream Regulator Analysis in IPA. The analytical tool is based on prior knowledge of expected effects between transcriptional regulators and their target genes stored in the Ingenuity® Knowledge Base.

Since miRNAs are not the only Dicer1- dependent factors that could trigger gene expression changes observed in the distal region of Dicer1 cKO mice, we performed a more exhaustive *in silico* analysis on whole transcript microarrays by using dedicated bioinformatics tools (*i*.*e*. Ingenuity Pathway Analysis (IPA), GSEA, ClueGO). Several pathways or functions have been highlighted in the distinct epididymal regions.

#### Immune dysfunction (corpus)

According to IPA analysis performed on genes with significantly changed expression levels in the corpus epididymidis (fold-change > 1.5, p-value < 0.01), 18 networks encompassing 4 or more modulated genes were identified from 426 probe-sets. Among the top-10 networks (S5 Fig), candidates associated with inflammatory functions and defence mechanisms were prominently associated with this epididymal region. These biological pathways were also confirmed by other bioinformatics programs such as GSEA and ClueGO. The latter identify inflammatory and immune defence responses as the most significantly altered functions in the corpus epididymidis of Dicer1 cKO mice. In addition, network analysis performed with more stringent parameters (fold-change > 2 and p-value < 0.001) on 24 probe-sets, sheds light on the alteration seen in the Dicer1 cKO epididymides with the “lipid metabolism, small molecule biochemistry, cell-to-cell signalling and interaction” networks highlighted (S6 Fig). One of these networks involves Pate4, phosphatidylethanolamine, and phosphatidylserine molecules.

#### Epigenetics regulation (corpus)

Upstream Regulator Analysis (URA) performed on our datasets, identified molecules located upstream of the changed genes to potentially explain the observed expression changes. We focused our study on the transcription factors and miRNAs that are potential modulators of gene expression in our model. According to this analysis, the corpus epididymis is significantly enriched with upstream regulators (UR) with elevated z-scores. Among the most significant URs, we identified 4 regulators associated with epigenetic control (*i*.*e*. histone deacetylases 1 and 2 (HDAC1 and HDAC 1), lysine (K)-specific demethylase 5B (KDM5B), and tripartite motif-containing 24 (TRIM24)). Furthermore, we identified 25 miRNAs with a significant UR potential (z-score > 2), including *miR-200* and *miR-204*–two miRNAs predicted to target and regulate the *Pate4* expression level. As shown in Fig 2E, *miR-204* is present in EVs derived from DC2 epididymal cells *in vitro*.

#### Sperm motility and physiopathologies of the male reproductive system (cauda)

**Fig 6:**
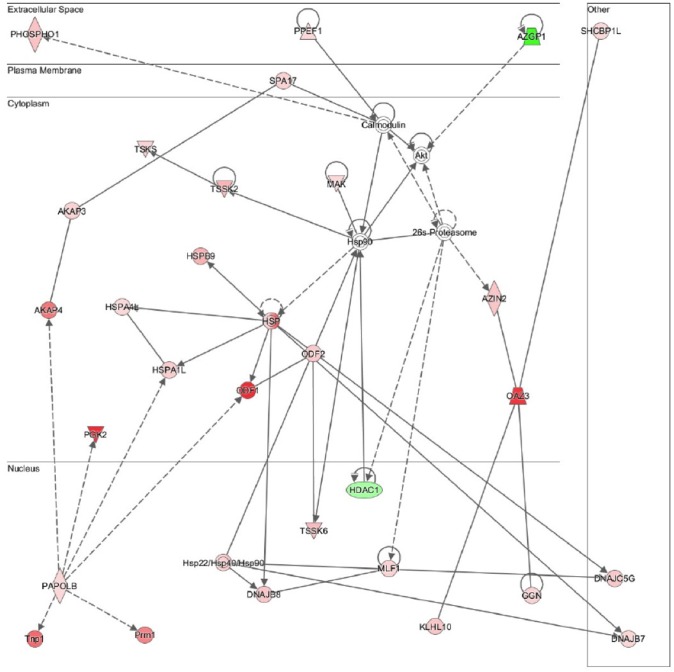
The “Reproductive system disease” network is the most significantly modified in the cauda epididymis of Dicer1 CKO compared to control mice according to Ingenuity Pathway Analysis (IPA) analysis. Only probe-sets displaying a fold change >1.5 and a p-value <0.01 were considered.

Among the top ten networks that are significantly modified in the cauda epididymidis of Dicer1 cKO compared to control mice (S7 Fig), “Reproductive system Disease” pathway displays the highest significance score, and encompasses a total of 29 transcripts. Interactions between the molecules involved in this pathway are displayed in Fig 6 and include AZGP1, which is shown as a regulator of downstream Akt signalling pathway. In addition, male reproductive and sperm motility dysfunctions display an important redundancy among pathways modified in the cauda epididymidis (not shown), which is in accordance with the reduced sperm motility and male infertility phenotypes observed in the Dicer1 cKO mouse model [5, 34].

## Discussion

In most–if not all–biological fluids, EVs and their cargo participate in well-orchestrated mechanisms of intercellular communication to control organ/systems physio-pathological conditions [8, 52, 53]. While such a long-distance signalling process is challenging to assess in the circulatory system, the epididymis represents an ideal enclosed and well-regulated model system to decipher the role of extracellular factors *in vivo*. In the present study we investigated the role of Dicer1-dependent factors, including miRNAs, in the paracrine regulation of epididymal genes by using the previously described *Defb41*^*iCre/wt*^*;Dicer1*^*fl/fl*^ (Dicer1 cKO) mouse model [5, 34].

In this genetically modified mouse model, the enzyme Dicer1 has been conditionally inactivated in the principal cells of the initial segment/caput epididymidis. This allowed the detection of 114 Dicer1-dependent miRNAs that have matured in this specific cell type, as they are significantly reduced or absent in the Dicer1 cKO *vs*. control. However, since miRNAs are ubiquitously present in whole epididymis extracts, *i*.*e*. in sperm cells and somatic cells from both the epithelium and the connective tissue [54, 55], it is possible that we have underestimated the number of Dicer1-dependent miRNAs produced specifically by principal cells. Furthermore, whereas miRNA maturation predominantly occurs via a Dicer1-mediated canonical process, other Dicer1-independent routes of maturation exist and are not considered in our approach [56]. Despite these limitations, 9 mature miRNAs exhibited significant and markedly reduced expression levels, including *miR-204*, a miRNA found particularly enriched in the proximal mouse epididymis [55] and *miR-138-5p*, a potential tumor suppressor that inhibits *cyclin D1 (Ccnd1)* expression [57].

As one miRNA can target up to several hundred mRNAs for degradation [2], we expected that Dicer1-dependent decrease/suppression of several miRNAs in the epididymis could have a profound impact on gene expression levels. Indeed, our results demonstrated significant region-specific changes in genes such as *nitric oxide synthase 1 (Nos1)* and *testis expressed 13A (Tex13a)* that usually show consistent levels of expression along the epididymis of wild type mice. This suggests that Dicer1–dependent factors derived from the proximal epididymis, could control gene expression levels in the distal epididymal regions in a paracrine-like manner. While it is tempting to link impairment of miRNA maturation with gene expression changes, we cannot exclude that other Dicer1-dependent factors, such as somatic endogenous small interfering RNAs (endo-siRNAs) may be involved in post-transcriptional gene regulation [58–60]. To assess the contribution of miRNA solely or miRNAs/endo-siRNA in this paracrine gene regulation, a comparative analysis of Dgcr8 and Dicer1 cKO mice would be required [58]. Furthermore, according to studies showing the role of DICER1 as a mRNA and long non-coding RNA binding protein as well as a nuclear factor in charge of heterochromatin formation [61, 62], its direct or indirect effect on miRNA-independent gene expression is also plausible.

Dicer1 deletion also triggered significant enrichment of some miRNAs in the IS/caput epididymis. Considering that 1) deletion of Dicer1 only occurs in the principal cells of the epididymis and that 2) whole epididymis extracts were used in our study, Dicer1 is still present/active in other epithelial cell types and stromal cells. As a consequence, some Dicer1–dependent miRNAs may be over-represented in Dicer1 cKO compared to control mice. A compensatory mechanism from Dicer1 expressed in adjacent tissues (*e*.*g*. efferent ducts) is also possible [5]. The explanation for the miRNA enrichment observed in Dicer1 cKO might therefore be a combination of these factors.

Since principal cells are specialized in the secretion of EVs called epididymosomes that transport a wide array of small non-coding RNAs [28, 30], we hypothesized that Dicer1-dependent miRNAs could be secreted via EVs and internalized by downstream target cells to control gene expression. To assess this hypothesis, we investigated the presence of Dicer1-dependent miRNAs in EVs derived from epididymal principal cells (DC2 cells) *in vitro*. We first showed that DC2 cells secrete EVs in the 90–400 nm size range that possess markers associated with distinct EV populations and/or functions. For instance, as previously shown in the epididymal fluid [27, 36], populations of DC2-derived EVs expose the CD9 antigen, a marker of exosomes [63], and phosphatidylserine (PS), a phospholipid also present on exosomes and microparticles. Exposure of PS on EVs mediates intercellular communication through binding to milk fat globule epidermal growth factor 8 (MFG-E8/SED-1) localized on recipient cells [64]. This glycoprotein is localized on the sperm surface and on epididymal epithelial cells and participates in epididymal fluid homeostasis [65–67]. Even if it has to be kept in mind that immortalized cell lines do not behave alike primary cells [68], DC2 cells share common features with principal cells of the epididymis regarding EV and miRNA production. For instance, DC2-derived EVs possess recognition molecules known to be important to intercellular communication, and DC2 cells express some miRNA candidates also found in epididymal principal cells (*e*.*g*. miR-210, miR-672, miR-191, miR-204). These miRNAs being secreted via DC2-derived EVs, they constitute potential candidates in the paracrine regulation of epididymal gene expression.

The comprehensive bioinformatics analysis of these microarray data indicated a potential association between proximal Dicer1-dependent miRNAs and distal gene expression /biological pathways (Table 2). For instance, *miR-204* is a secreted miRNA predicted to target the *Pate4* transcript and other associated genes that are involved in the control of sperm motility. *Pate4* belongs to a gene family that is predominantly expressed in the epididymis and is proposed to interact with spermatozoa to modulate their motility, capacitation and acrosome reaction [69, 70]. Therefore, *Pate4* and *miR-204* are interesting targets that could–at least in part–explain the reduced motility phenotype observed in Dicer1 cKO mice [34]. In addition, we showed that AZGP1 is a glycoprotein highly expressed in a regionalized manner along the mouse epididymis, and that is localized on the sperm equatorial region. Our results indicate that AZGP1 expression is significantly increased in the cauda epididymidis of Dicer1 cKO at both the transcriptional and protein levels. This protein participating to the control of sperm forward motility [71], its deregulation in the epididymis by Dicer1-dependent factors including miR-296 might also have consequences on sperm motility. Both PATE4 and AZGP1 being proteins secreted into the extracellular environment, they might thus constitute appealing targets for the non-invasive diagnosis of male infertility.

In conclusion, epithelial cells of the epididymis are specialized cells that communicate with each other in order to control the environment surrounding sperm during their maturation. In our study, we showed that Dicer1-dependent factors from the proximal epididymis, including miRNAs, (1) are released from principal cells of the epididymis and (2) impact, either directly or via alteration of pronounced changes in epididymal functions (*e*.*g*. epithelial dedifferentiation, abnormal lipid homeostasis), the profiles of epididymal gene expression in the downstream regions. While further *in vivo* studies will be required to unravel the complete extracellular vesicular and miRNA cargo signalling system in the epididymis, our study sheds light on new molecular candidates that may be important in the control of male fertility.

## Supporting Information

S1 Fig RNA integrity assessment. RNA integrity numbers were obtained with the Agilent 2100 Bioanalyzer for all samples used in the present microarray study from control (Ctrl) and Dicer1 cKO (cKO) mice.
(TIF)

S2 Fig Microarray quality control plots for miRNAs (A) and whole transcript (B).
(TIF)

S3 Fig Quantitative dynamic range assessment of AZGP1 by western-blot. (A) Different protein concentrations from mouse epididymal extracts were loaded and blotted for AZGP1 and Beta-actin. (B) Protein band volumes were measured and plotted to assess protein dynamic ranges.
(TIF)

S4 Fig Segment-specific expression of transcripts and miRNAs in control and Dicer1-cKO mice. (A) According to microarray data, the defensin family in Control (Ctrl) and Dicer1 cKO mice follows the same segmented gene expression pattern as described in wild type mice (Johnston *et al*, 2005). For instance, Defb1,2,9,10 and 11 display a higher expression level in the cauda epididymis (Cau) of Ctrl and Dicer1 cKO mice compared to Defb13, 15, 19, 35 and rs1 that are more expressed in the corpus (Cor) epididymis. (B) Validation of Dicer1 expression in control and Dicer1 cKO mice epididymis by real-time PCR. Unpaired T-test; ****: P<10^−4^; ns: not significant. (C) Expression level of miR-204-5p and miR-196 in Ctrl mice follows the same pattern as in wild-type mice described in Nixon *et al*, 2015.
(TIF)

S5 Fig Top ten networks significantly modified in the corpus epididymis of Dicer1 cKO compared to control mice according to IPA analysis. Only probe-sets displaying a fold change >1.5 and a p-value <0.01 were considered. Total of probe-sets included = 426.
(TIF)

S6 Fig Top networks significantly modified in the corpus epididymis of Dicer1 cKO compared to control mice according to IPA analysis. Only probe-sets displaying a fold change >2 and a p-value <0.001 were considered. Total of probe-sets included = 24.
(TIF)

S7 Fig Top ten networks significantly modified in the cauda epididymis of Dicer1 cKO compared to control mice according to IPA analysis. Only probe-sets displaying a fold change >1.5 and a p-value <0.01 were considered. Total of probe-sets included = 513.
(TIF)

S1 Table Sequences and properties of primers used for qRT-PCR on target transcripts. (XLSX)

S2 Table Sequences and properties of primers used for qRT-PCR on target microRNAs. (XLSX)

S3 Table ANOVA results obtained from miRNA microarrays on Dicer1 cKO vs. control caput epididymidis. (XLSX)

S4 Table ANOVA results obtained from whole transcript microarrays on Dicer1 cKO vs. control corpus epididymidis. Transcripts with a fold change >1.5 or <-1.5 and P-value <0.01 are displayed.
(XLSX)

S5 Table ANOVA results obtained from whole transcript microarrays on Dicer1 cKO vs. control cauda epididymidis. Transcripts with a fold change >1.5 or <-1.5 and P-value <0.01 are displayed.
(XLSX)

S6 Table ANOVA results obtained from whole transcript microarrays on Dicer1 cKO vs. control corpus and cauda epididymidis. (XLSX)
