# Supplementary figures and images for: Role of Dicer1-Dependent Factors in the Paracrine Regulation of Epididymal Gene Expression
Source: PLoS One. 2016 Oct 3;11(10):e0163876. doi: 10.1371/journal.pone.0163876 (PMC5047620; doi:10.1371/journal.pone.0163876)

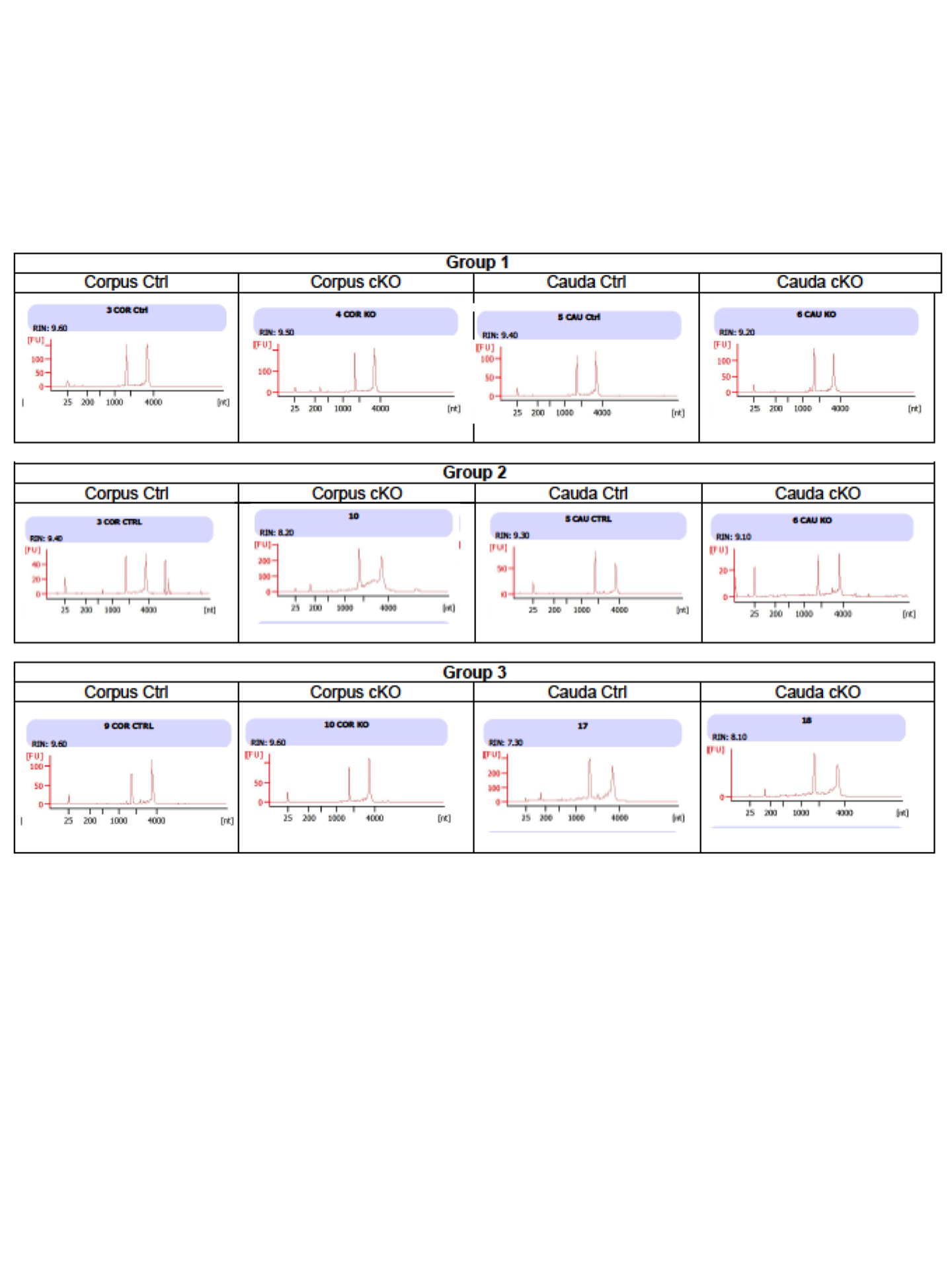

Supplement: S1 Fig — RNA integrity numbers were obtained with the Agilent 2100 Bioanalyzer for all samples used in the present microarray study from control (Ctrl) and Dicer1 cKO (cKO) mice. (TIF) [file pone.0163876.s001.tif]

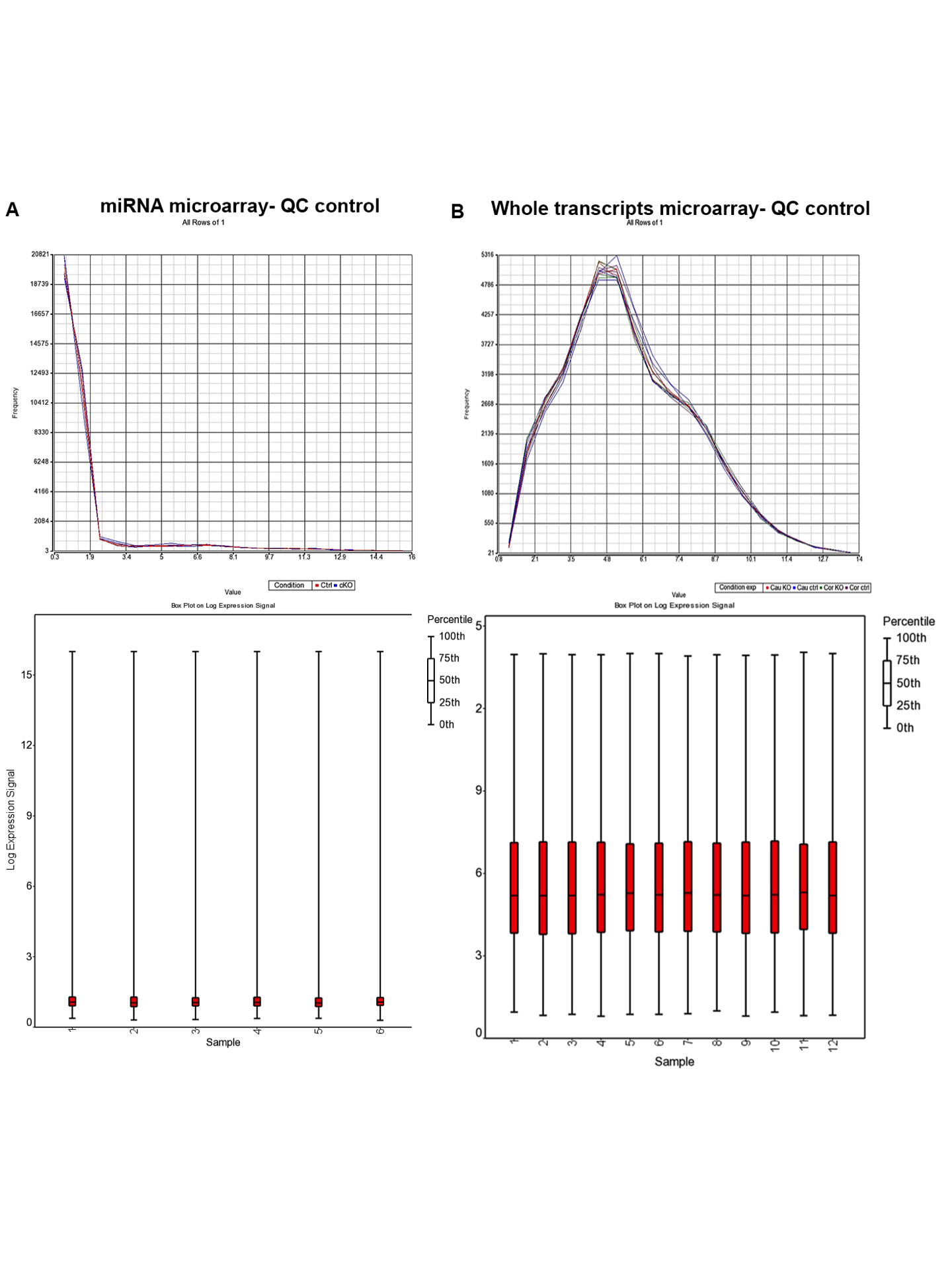

Supplement: S2 Fig — Microarray quality control plots for miRNAs (A) and whole transcript (B). (TIF) [file pone.0163876.s002.tif]

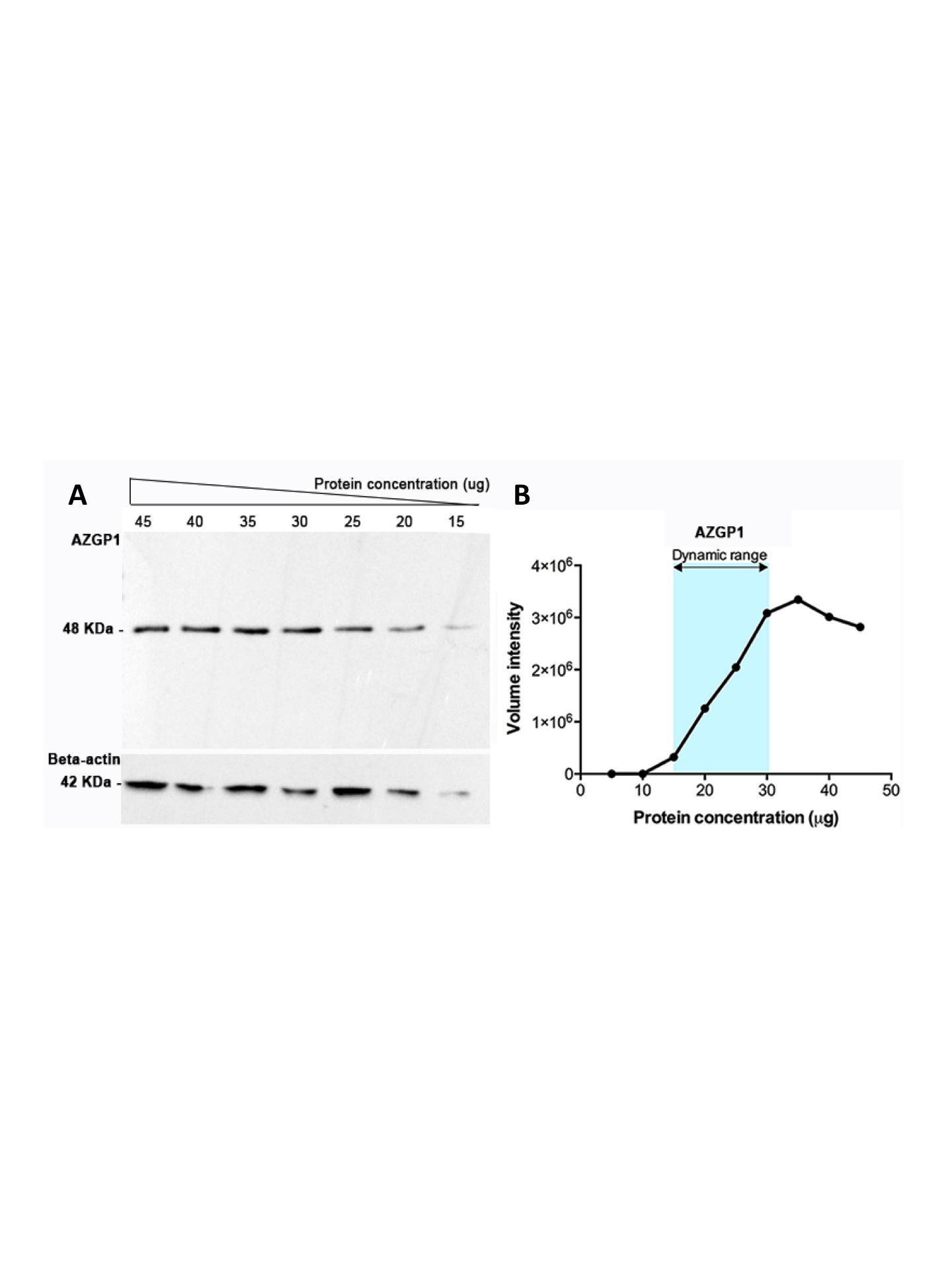

Supplement: S3 Fig — (A) Different protein concentrations from mouse epididymal extracts were loaded and blotted for AZGP1 and Beta-actin. (B) Protein band volumes were measured and plotted to assess protein dynamic ranges. (TIF) [file pone.0163876.s003.tif]

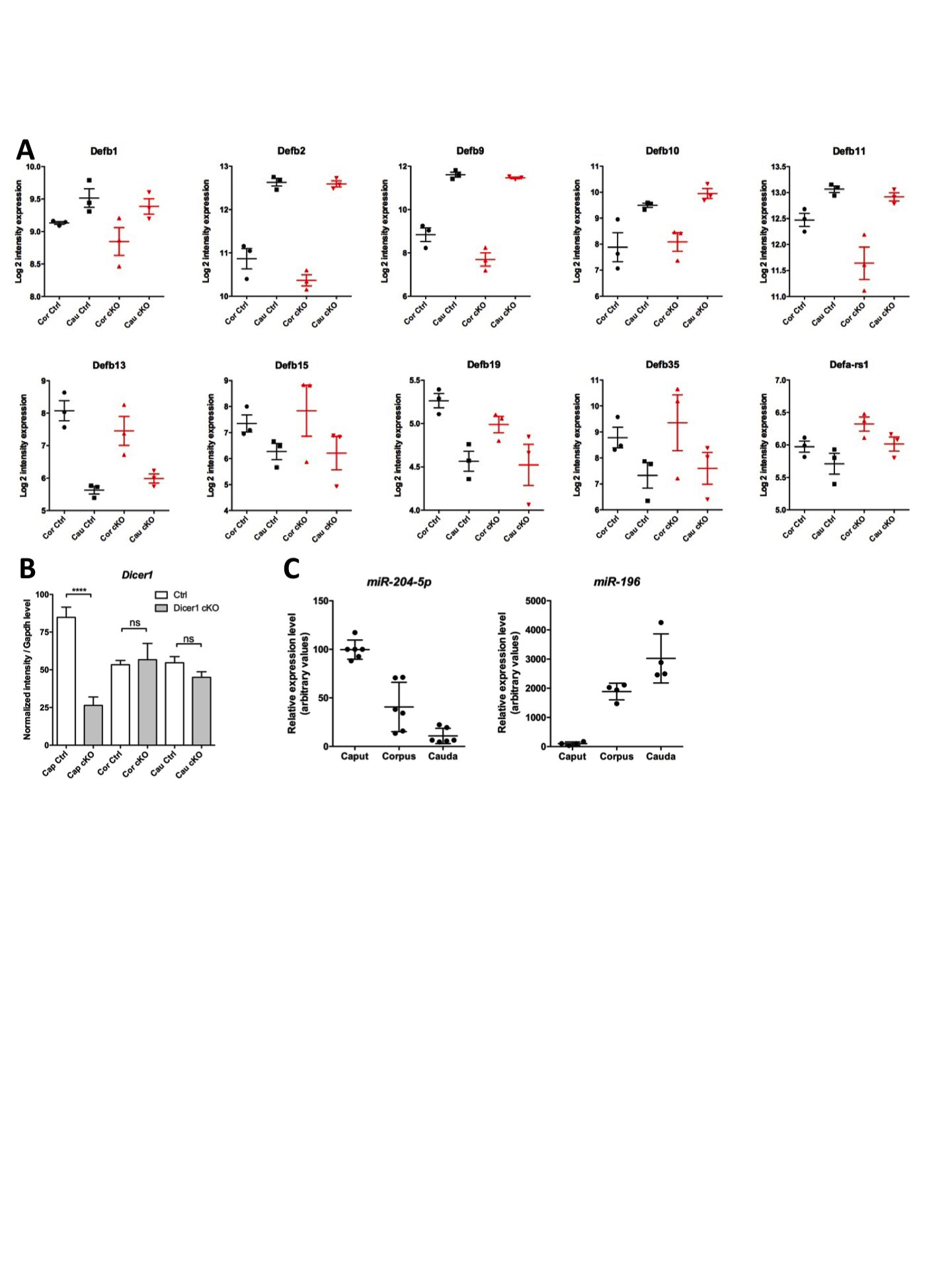

Supplement: S4 Fig — (A) According to microarray data, the defensin family in Control (Ctrl) and Dicer1 cKO mice follows the same segmented gene expression pattern as described in wild type mice (Johnston et al, 2005). For instance, Defb1,2,9,10 and 11 display a higher expression level in the cauda epididymis (Cau) of Ctrl and Dicer1 cKO mice compared to Defb13, 15, 19, 35 and rs1 that are more expressed in the corpus (Cor) epididymis. (B) Validation of Dicer1 expression in control and Dicer1 cKO mice epididymis by real-time PCR. Unpaired T-test; ****: P<10−4; ns: not significant. (C) Expression level of miR-204-5p and miR-196 in Ctrl mice follows the same pattern as in wild-type mice described in Nixon et al, 2015. (TIF) [file pone.0163876.s004.tif]

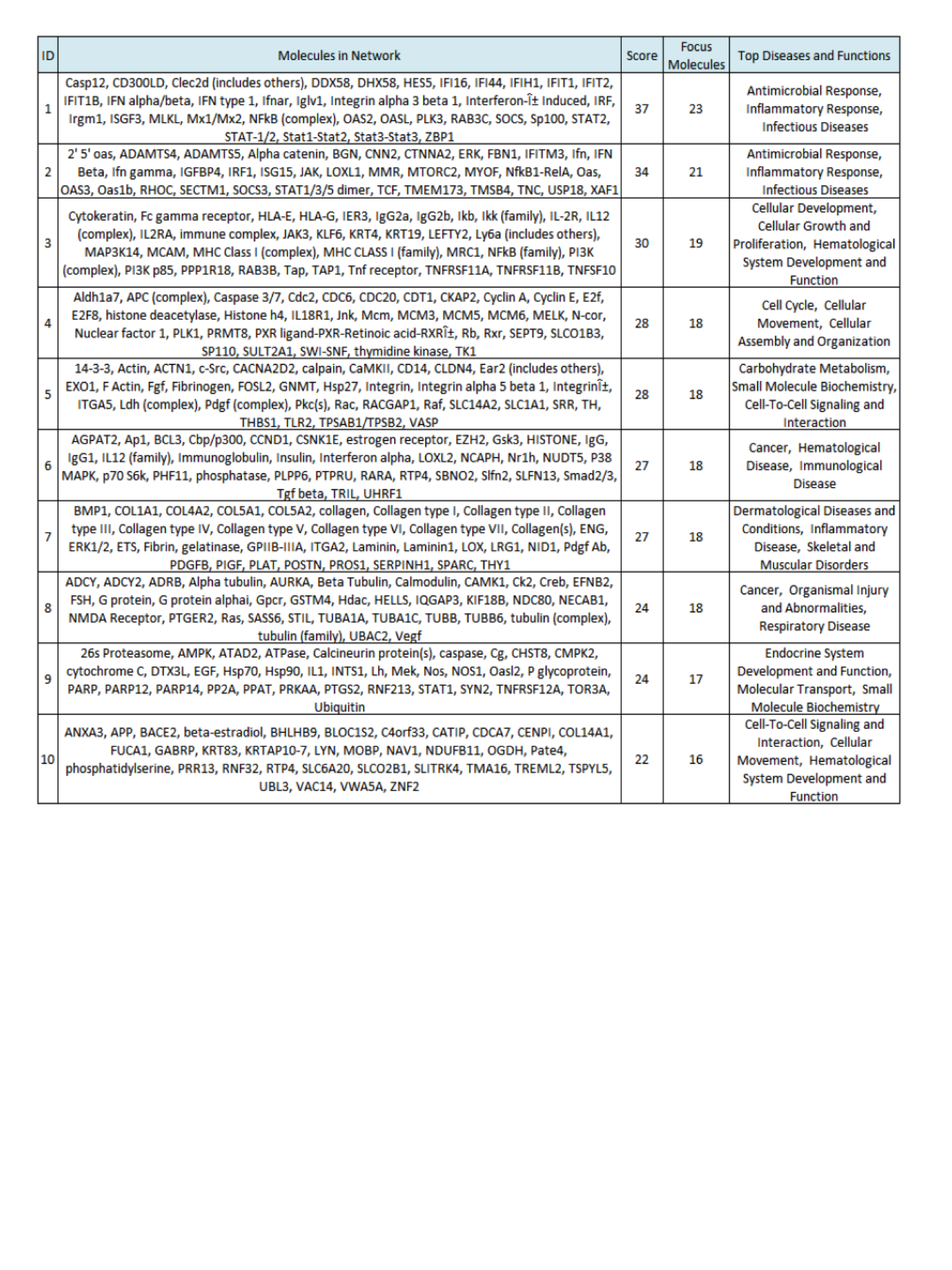

Supplement: S5 Fig — Only probe-sets displaying a fold change >1.5 and a p-value <0.01 were considered. Total of probe-sets included = 426. (TIF) [file pone.0163876.s005.tif]

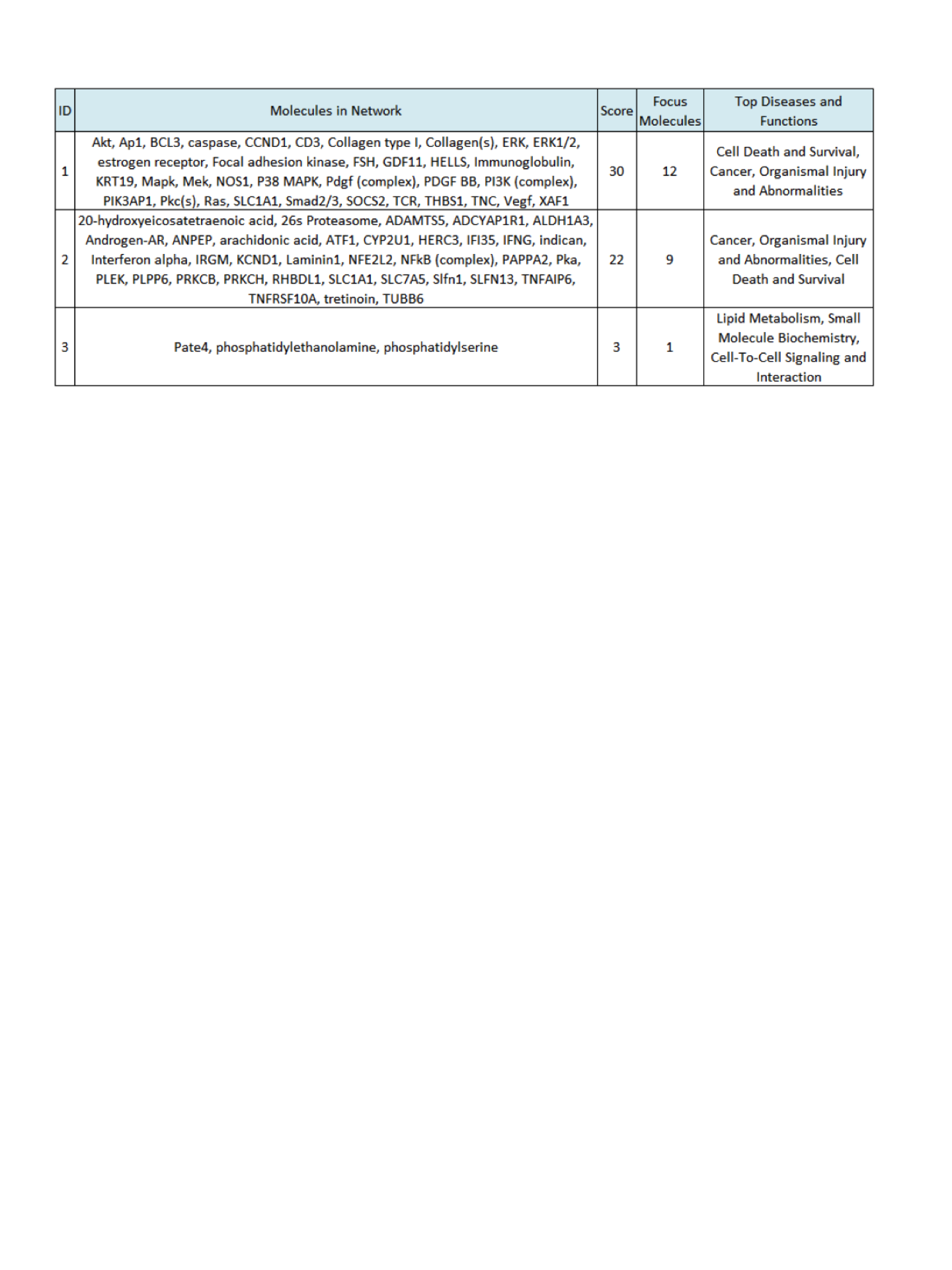

Supplement: S6 Fig — Only probe-sets displaying a fold change >2 and a p-value <0.001 were considered. Total of probe-sets included = 24. (TIF) [file pone.0163876.s006.tif]

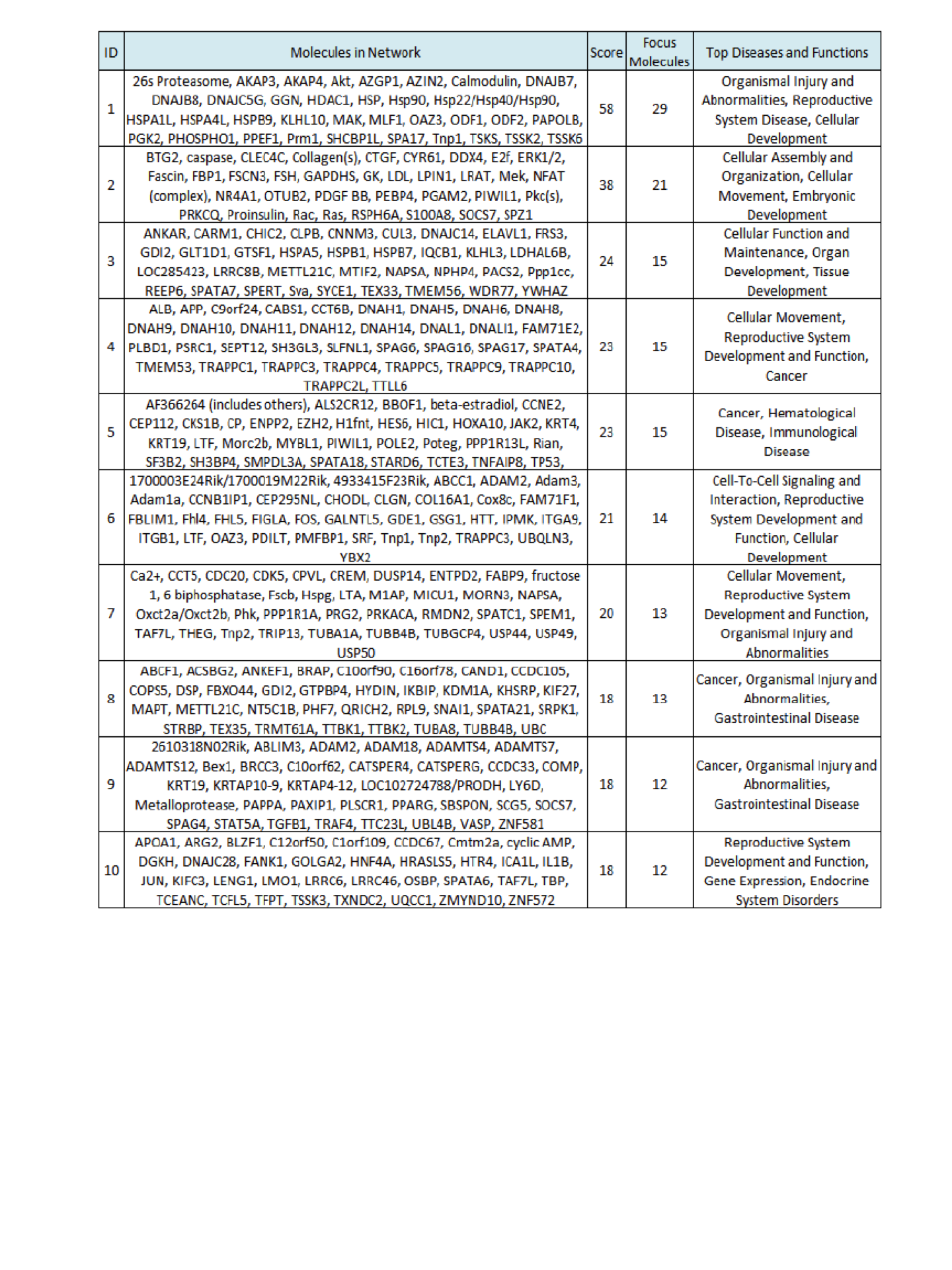

Supplement: S7 Fig — Only probe-sets displaying a fold change >1.5 and a p-value <0.01 were considered. Total of probe-sets included = 513. (TIF) [file pone.0163876.s007.tif]
